# Effects of removing woody cover on long‐term population dynamics of a rare annual plant (*Agalinis auriculata*): A study comparing remnant prairie and oldfield habitats

**DOI:** 10.1002/ece3.4654

**Published:** 2018-11-11

**Authors:** Helen M. Alexander, Cathy D. Collins, Aaron W. Reed, W. Dean Kettle, Daniel A. Collis, Lucy D. Christiana, Vaughn B. Salisbury

**Affiliations:** ^1^ Department of Ecology and Evolutionary Biology University of Kansas Lawrence Kansas; ^2^ Biology Program Bard College Annondale‐on‐Hudson New York; ^3^ School of Biological Sciences University of Missouri‐Kansas City Kansas City Missouri; ^4^ Kansas Biological Survey University of Kansas Lawrence Kansas; ^5^ Present address: 1623 E. 120th Street Olathe Kansas

**Keywords:** annual, oldfield, population dynamics, prairie, rare plant, survey, woody encroachment

## Abstract

Worldwide, grasslands are becoming shrublands/forests. In North America, eastern red cedar (*Juniperus virginiana*) often colonizes prairies. Habitat management can focus on woody removal, but we often lack long‐term data on whether removal leads to population recovery of herbaceous plants without seeding. We undertook a long‐term study (17 years) of numbers of the rare annual plant *Agalinis auriculata* in a gridwork of 100 m^2^ plots in adjacent prairie and oldfield sites in Kansas, USA. We collected data before and after removal of *Juniperus virginiana* at the prairie. Plant population sizes were highly variable at both sites and over time. High numbers of plants in a plot 1 year were often followed by low numbers the following year, suggesting negative density‐dependence. Plant numbers were lowest with extensive woody cover and with low precipitation. After woody plant removal, *A. auriculata* increased dramatically in abundance and occupancy in most years; increases were also seen at the oldfield, suggesting later survey years were overall more favorable. *Synthesis and applications*: Removal of woody plants led to increased numbers of a rare annual prairie plant, without seeding. Multiple years of data were essential for interpretation given extreme temporal variability in numbers. The largest prairie population was 7 years following tree removal, showing that positive effects of management can last this long. This species also fared well in oldfield habitat, suggesting restoration opportunities. Given that land managers are busy, time‐efficient field methods and data analysis approaches such as ours offer advantages. In addition to general linear models, we suggest Rank Occupancy‐Abundance Profiles (ROAPs), a simple‐to‐use data visualization and analysis method. Creation of ROAPs for sites before and after habitat management helps reveal the degree to which plant populations are responding to management with changes in local density, changes in occupancy, or both.

## 1 INTRODUCTION

Temperate grasslands are conservation priorities; these once vast ecosystems have a long history of transformation to agriculture. Grasslands around the world now face a new threat: conversion into shrublands or forests as a result of woody plant colonization. These changes are occurring rapidly and have profound implications for ecosystem properties and biodiversity (Briggs, Hoch, & Johnson, 2002; Knapp et al., 2008). Global (e.g., climate change, CO_2_ changes) and local (e.g., changes in fire or grazing regimes) drivers contribute to these ecosystem shifts (Bond & Midgley, 2012; Ratajczak, Nippert, Briggs, & Blair, 2014).

Studies at the local spatial scale are particularly relevant to land managers who want to know how management decisions affect grassland populations and communities. For example, removal of eastern red cedar (*Juniperus virginiana*) can sometimes lead to recovery of herbaceous communities (Alford, Hellgren, Limb, & Engle, 2012; Limb, Engle, Alford, & Hellgren, 2014; Pierce & Reich, 2010). These studies took a broad perspective, using many sites and taking data on percent cover or biomass of many species. Although invaluable from a community perspective, such data may lack adequate resolution for species‐specific investigations. Yet ultimately the question of community transformation or recovery depends on the population ecology of individual species: that is, are increases or decreases of woody cover associated with predictable growth or decline of herbaceous plant populations? Dedicated population approaches are particularly needed for rare species that may not occur regularly in small sampling plots.

From a population ecology perspective, two complementary approaches are available: demographic studies and long‐term surveys of plant numbers. The former consists of following plants to document survival and reproduction and developing demographic models (Caswell, 2001). For example, Andrieu et al. (2013) found that reproduction and asymptotic population growth rates of a rare perennial increased greatly after forest cutting and were comparable to open‐habitat populations. Demographic models, however, often fail at forecasting dynamics of future years (Crone et al., 2013). This result is not surprising: environments change over time and these models are not designed to take into account all factors, including density‐dependence.

In contrast to demographic models, long‐term survey data document actual population dynamics—did a population decline in numbers with increased woody cover, and increase when woody plants were removed? Recording data in a continuous gridwork of plots allows analyses of both abundance and occupancy, and is important since plants in future years may occur in parts of the site where they are not currently found (Crawley, 1990). Survey data are most useful if the plant's life cycle is short relative to the data set length. It is thus not surprising that many studies of annual population dynamics have been done (Garcia de Leon, Freckleton, Lima, & Navarrete, 2014; Plaza, Navarrete, Lacasta, & Gonzalez‐Andujar, 2012). Annual plants are challenging to study, however, because of yearly fluctuations in numbers, which can be due to processes originating within (endogenous) or outside (exogenous) of the population (Plaza et al., 2012). A common endogenous process is negative density‐dependence (Garcia de Leon et al., 2014; Gonzalez‐Andujar, Fernandez‐Quintanilla, & Navarrete, 2006). Typical exogenous processes are temperature and precipitation (Garcia de Leon et al., 2014; Levine, Mceachern, & Cowan, 2011). Annual plants also often have seed banks, and germination of buried seed from past years may be important in population dynamics (Alexander, Pilson, Moody‐Weis, & Slade, 2009; Salguero‐Gomez, Siewert, Casper, & Tielborger, 2012).

**Figure 1:**
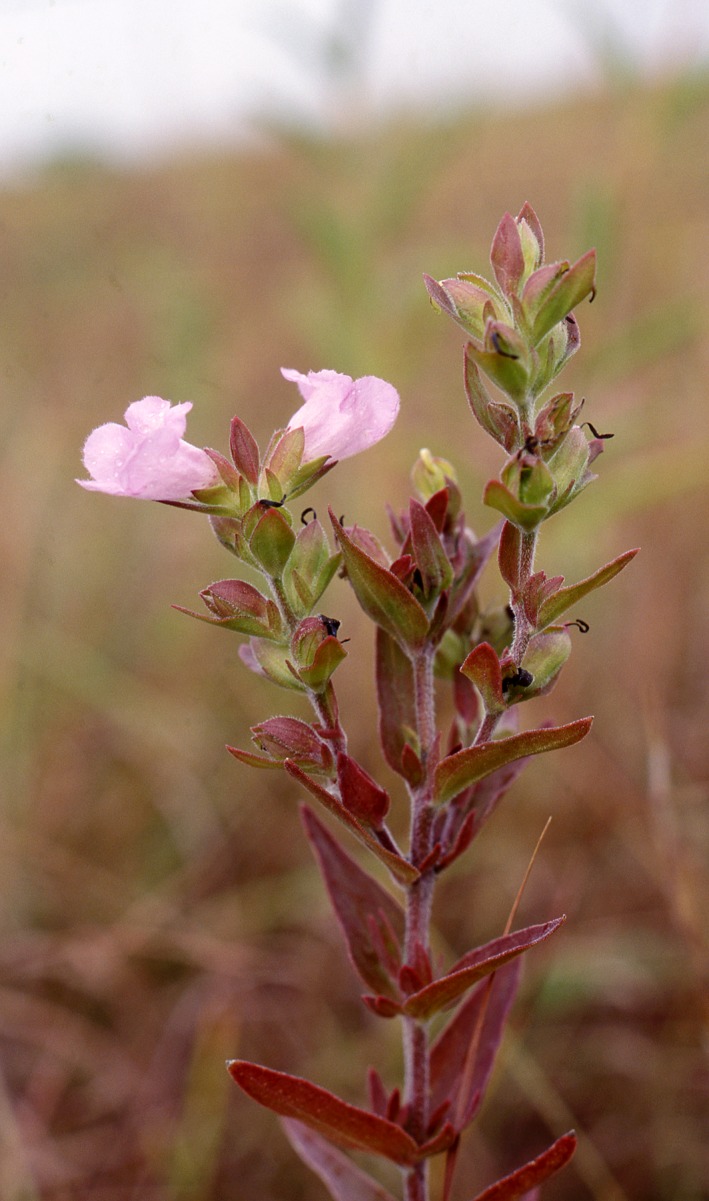
Flowering individual of *Agalinis auriculata*. Photograph by Craig Freeman

In North American tallgrass prairies, the annual life form is uncommon: most plants are perennial. Our work examined the rare annual *Agalinis auriculata *in Kansas, USA in the context of woody colonization by eastern red cedar (*Juniperus virginiana*; Figure 1). This tree has increased across central North America (Meneguzzo & Liknes, 2015), primarily due to fire suppression (Ratajczak et al., 2014). As woodlands expand, grassland habitats decline: Briggs et al. (2002), for example, found fewer than five species/plot in high‐density cedar regions compared to 20–30 species in plots without cedar. Demographic effects are also apparent: Albrecht, Becknell, and Long (2016) noted that reproduction of the perennial *Astragalus bibullatus* declined in areas with cedar colonization.

Past work suggests that woody species have negative effects on *A. auriculata*. In Ohio, the largest populations were in areas with tree and shrub removal (Knoop, 1988). Based on a 4‐year study in Illinois, Vitt, Havens, Kendall, and Knight (2009) concluded that populations should decline in the presence of woody brush (lambda = 0.81) and grow (lambda = 1.22) with brush removal. They cautioned that the positive effects of management on *A. auriculata* may be temporary, since competitors (often grasses) also increase after woody removal.

We began studying *A. auriculata *in 1996 at a remnant prairie, and expanded our work in 1997 to include an adjacent oldfield. In 2006, we removed *J. virginiana* and other woody species at the prairie with the goal of improving habitat for *A. auriculata. *Data collection continued through 2013, making it one of the longest data sets for terrestrial annuals outside of agriculture (Plaza et al., 2012). We first tested the hypothesis that woody plant presence alters abundance of *A. auriculata*. Second, we tested Vitt et al.’s (2009) hypothesis that positive effects of woody removal may be of short duration. Third, we addressed the degree to which *A. auriculata *is dependent on remnant prairie: were numbers comparable in the oldfield and prairie? With both sites, we also explored how other factors were associated with plant numbers, ranging from precipitation to local variation in past plant numbers.

## 2 METHODS

### 2.1 Study organism and sites

*Agalinis auriculata* (Michx.) S. F. Blake (Orobanchaceae, formerly *Tomanthera auriculata*) historically occurred across eastern United States in prairies and other open sites (Pennell, 1935). It currently has a limited distribution and was considered for listing under the Endangered Species Act (U.S. Fish & Wildlife Service, 1993).

Although *A. auriculata* photosynthesizes, it is hemiparasitic (or facultative hemiparasitic), with greenhouse studies showing haustoria development occurs with plants in the Asteraceae such as *Helianthus occidentalis, Silphium terebinthinaceum, *and *Solidago rigida *(Molano‐Flores, Feist, & Whelan, 2003). Seeds germinate in the spring and flowers (1.5 cm long) are produced in the fall. Plants are self‐compatible but outcrossing also likely occurs (Molano‐Flores et al., 2003; Mulvaney, Molano‐Flores, & Whitman, 2004). Seeds require light for germination and persist in the soil for at least 3.5 years (Baskin, Baskin, Parr, & Cunningham, 1991). In Illinois, there was a 14‐fold variation in plant numbers over a 4‐year period (Mulvaney et al., 2004).

**Figure 2:**
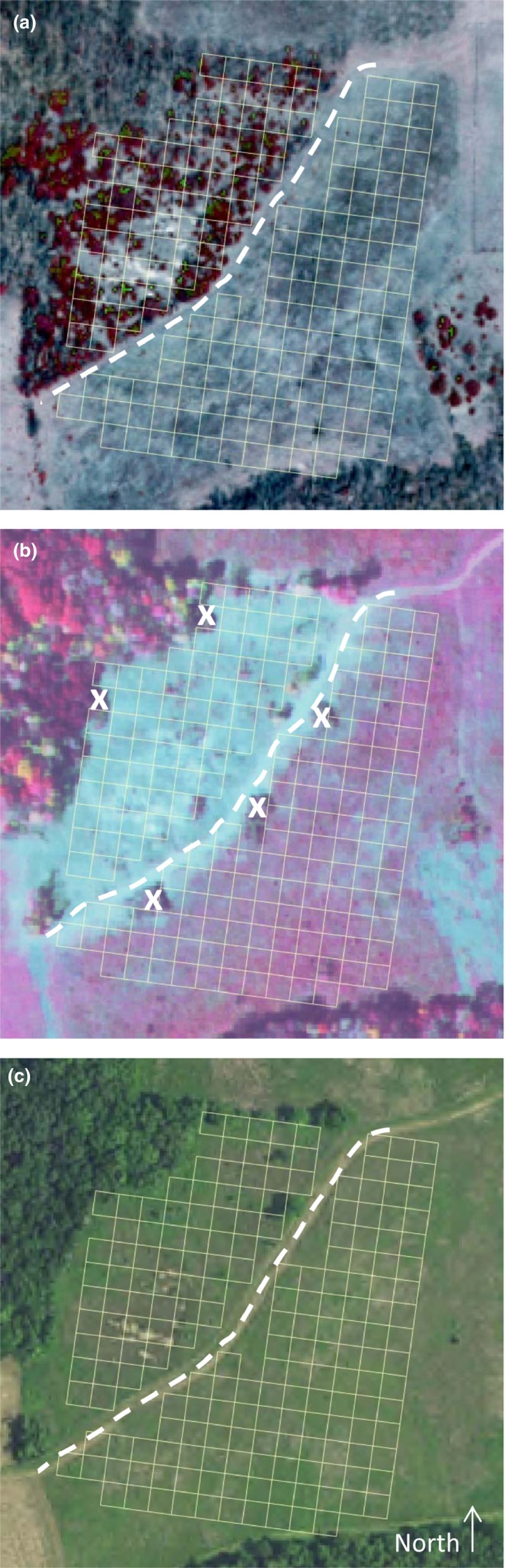
Aerial photographs with grid (100 m^2 ^plots) before and after woody removal. The path (dashed line) separates the prairie (66 plots, north of path) and oldfield (119 plots, south of path). (a) 2002 color infrared image, before vegetation management; (b) 2006 color infrared image immediately after woody removal on the prairie; “X’ marks locations where cut trees/brush were piled; (c) 2015 color image after all management, including burning and mowing. Plots on the path or that had tree/brush piles on them after woody removal were not used in most analyses; see Section 2.3. Light‐colored patches of eroded soil are apparent in 2002 and 2015 prairie images.

Our study was conducted at adjacent sites at the University of Kansas Field Station (10 km north of Lawrence, Kansas, USA, 39.055455N, 95.200519W; Figure 2). *Agalinis auriculata* was first discovered at the 8,200 m^2^ tallgrass prairie site in 1989 and Ward (1994) noted ~150 plants in 1991–1992. A high‐quality 1971 photograph shows a few scattered trees in the prairie and no trees in the oldfield. Since 1971, colonization of eastern red cedar (*Juniperus virginiana *L. (Cupressaceae)) is increasingly evident in photographs. Aerial photographs of the prairie dating back to 1937 also reveal several patches of severely eroded soil with sparse vegetation (total area 1,600 m^2^). A 13,900 m^2^ oldfield is adjacent to the prairie (separated by a 3 m path). *Agalinis auriculata* were first noticed in the oldfield in 1991, ~30 m from the prairie. The oldfield has a history of tillage (most recently in 1995) and virtually no woody colonization.

In fall 2006 (after surveys, see below), all woody vegetation (except small shrubs) was cut in the prairie at ground level. Over 90% of the cut plants were *J. virginiana. *See Supporting information Appendix S1 for study site details.

### 2.2 Data collection

#### 2.2.1 Plant surveys

In 1996, a 10 m by 10 m gridwork was overlaid on the prairie, producing 100 m^2^ plots (Figure 2). In late August—early October from 1996–2013 (excluding 2011), we counted the number of plants of *A. auriculata* (nearly all were flowering) in plots, typically by a person marking each plant with a flag, and then counting flags. In dense areas, survey counts (counts = number of plants) were based on intensive counting in subsets of the plot, followed by estimating numbers per plot. Our analyses focus on the 66 prairie plots that did not intersect the path.

Similar surveys were conducted in the oldfield. Initially (1997–2000), number of plants were only consistently counted in six 100 m^2^ plots just east of the path. In 2001, the gridwork was expanded to cover the entire oldfield; these 119 plots were counted from 2001 to 2010 (Figure 2). No data were recorded in 2011 and 2012; in 2013, very large numbers of plants were present so counts were only done in 14 plots.

The same people (especially VBS) directed surveys each year, resulting in consistent methodology.

#### 2.2.2 Precipitation

Precipitation data (standard can measurements) were available as monthly summaries from a Field Station climate monitoring site 1 km southeast of the sites (https://biosurvey.ku.edu/field-station ). We analyzed annual precipitation (cm) using a 12‐month period beginning October 1 of a given year and ending September 30 of the following year. The 2000 value, for example, includes 1999–2000 fall/winter precipitation (affecting seed overwintering) and 2000 growing season precipitation (affecting seed germination and plant growth), both of which may affect numbers in fall 2000.

#### 2.2.3 Woody cover and eroded soil

We created a georeferenced shapefile of the grid using ArcMap10.3.1. This grid was overlain on rectified aerial photographs to permit visual estimation of woody and eroded soil cover.

We had aerial photographs from 1991 (USGS Digital Orthophoto Quadrangle), 2002, 2004, 2005, 2006, 2008, 2010, 2012, and 2015 (Farm Service Agency, NAIP), and from special missions undertaken in 2002 and 2006 producing color infrared (CIR) images. Our visual estimates of woody cover per 100 m^2^ plot from 1996 to 2013 came from 1991, 2002 CIR, 2006 CIR, and 2015 photographs (and historical photographs, including Google Earth). If photographs were not available, we interpolated cover using estimates available from preceding and later years.

We estimated percent eroded soil/plot using a 2012 image.

#### 2.2.4 Data sets

We created two types of data sets. First, to explore dynamics over the longest period of time, we analyzed the average number of plants per plot per year (average plant data set). For the prairie, we calculated this variable by summing all plants seen in a year and dividing it by the number of plots (66; such values are available 1996–2013 [excluding 2011]). For the oldfield, we took this same approach for 2001–2010 (summing all plants seen in a year and dividing by 119, the number of plots); see Supporting information Appendix S1 for calculation details for other years where <119 plots were surveyed. For both sites, we excluded plots along the path or that had woody brush piles in some years. The average plant data set had 17 (prairie) or 15 (oldfield) data rows (1 row per year).

Second, we created a local data set to address plot‐to‐plot variation and to consider possible effects of neighboring plots. For the prairie, the local data set had 66 rows, corresponding to the 66 plots surveyed from 1996 to 2013 (excluding 2011). The oldfield local data set had 119 rows (119 plots surveyed from 2001 to 2010). These local data sets did not include plots along the path or those that had woody brush piles in some years. For each plot in both local data sets, we summed the number of plants in all 8 plots that surrounded it. Such “neighbor plants” could be a source of seed dispersal and thus contribute to numbers in a plot in the subsequent year. In counting neighbors, we included plots that were on the path or ones with woody brush piles since such plots had been surveyed for plants in all years except 2013. If a plot was on the gridwork edge, it had fewer than 8 neighbors; this was not problematic since the gridwork area covered the area where *A. auriculata *was abundant and we expected no (or only a few) plants outside the gridwork.

### 2.3 Analyses

#### 2.3.1 Overview

With the exception of Rank Occupancy‐Abundance Profile (ROAPS) analyses (see below), our goal was to explore whether variation in plant abundance within or across years was associated with exogenous factors (e.g., precipitation in a current [*t*] or previous year (*t* − 1); percent woody cover and percent eroded soil in the current year [*t*]) and endogenous factors (e.g., number of plants present in the plot the previous year (*t* − 1) and number of neighbor plants in both the current year *t* and previous year *t* − 1). Details are described below; in all cases, we first fit a global model and compared the fit of models with fewer parameters to the global model. We used Akaike's information criterion (AIC) values to select the most parsimonious models from the set of explored models. If a variable was kept in the model, the most important feature is the sign of the coefficient (i.e., if positive, that variable was associated with increased plant numbers). Following Burnham and Anderson (1998), the best model had the lowest AIC score and we considered models with a ΔAIC > 2 to have more support than other competing models and used parameter estimates derived from that model. We used SAS (version 9.4).

#### 2.3.2 Average number of plants/plot across years

We used multiple linear regression to determine what variables best predicted the average number of plants/plot. We used the average plant data sets in separate analyses for the prairie (1996–2013, excluding 2011) and oldfield (1997–2013, excluding 2011, 2012). Variables in the initial model for each site were precipitation_t_, precipitation_t‐1_, number of plants_t‐1_, number of neighbors_t_, and number of neighbors*_t_*_−1_. Average number of plants per plot was log‐transformed to satisfy the assumption of normality in linear regression.

#### 2.3.3 Number of plants/plot: within years (prairie)

We also determined which variables best predicted plant numbers within a year. We used log‐linear models in single year analyses with only the local prairie data set. We analyzed years before (1997, 1999, 2001) and after (2008, 2009, 2010, 2012, and 2013) woody removal (only years where at least one plot had>5 plants). Variables included in initial models were percent woody cover_t_, percent eroded soil_t_, number of plants_t‐1_, number of neighbors_t_, and number of neighbors*_t_*_−1_. We compared fit of zero‐inflated models with Poisson models (not zero‐inflated). Models for 2009 and later were best described by a log‐linear model with a Poisson distribution. Models before 2009 were best fit with a zero‐inflated Poisson.

#### 2.3.4 Comparison: 2001–2006 versus 2007–2010 (plants/plot)

Using the local data sets, we ran four models: both the prairie and oldfield sites for 2001–2006 (before woody removal in the prairie) and for 2007–2010 (after woody removal in the prairie). Woody cover did not change over time at the oldfield, but we compared both sites for the same periods to evaluate if factors other than woody removal were changing plant populations. Variables included in the initial model were precipitation_t_, precipitation_t‐1_, number of plants_t‐1_, number of neighbors_t_, and number of neighbors _t‐1_. We used a zero‐inflated model with mixed effects to fit regressions (plot was a fixed effect; year was a random effect). We compared fit among possible distributions (negative binomial or Poisson) by comparing the predicted number of zeros with the observed. All models were best fit by a zero‐inflated Poisson model.

#### 2.3.5 Comparision: 2001–2006 versus 2007–2010 (abundance and occupancy)

We used the local data sets (prairie and oldfield) and Rank Occupancy‐Abundance Profile (ROAP) analyses (Collins, Holt, & Foster, 2009) to explore temporal changes in local abundance (number of plants/plot) and occupancy (proportion of plots occupied). In a ROAP plot, the *y*‐axis displays local abundance in each plot, and the maximum value represents the plot with the highest abundance. Plots are ranked in order of their abundance along the *x*‐axis, with the highest abundance plot being ranked first (“1”). When the rank is divided by the number of plots surveyed, the maximum *x*‐value displays the proportion of plots occupied in the landscape (i.e., occupancy). A ROAP plot for a year allows one to quickly visualize the maximum abundance (*y*‐intercept), the range of abundances across the plots (other y values), and the occupancy (*x*‐intercept). Total abundance is the area under the ROAP; the area between two ROAPs displayed on the same graph reflects magnitude of change in overall abundance, accounting for both spatial expansion or retraction, as well as local plot densities. See Supporting information Appendix S2 for example data sets and ROAPs.

We constructed ROAPs for both prairie and oldfield for each year. To reduce the number of statistical comparisons and to focus on our research questions, we also constructed average ROAPs for two times: before woody removal (*T*_1_), and after woody removal (*T*_2_). We made two comparisons: (a) using the same years where data were available in both prairie and oldfield (before, 2001–2006 vs. after, 2007–2010) and (b) using all data (prairie only; before, 1996–2006 vs. after 2007–2013, excluding 2011). The former allowed us to explore if factors other than woody plants were altering population dynamics. Averages were calculated for each rank over the appropriate time periods on data sets where each year had been sorted by plot abundance. In plots of average ROAPs, the *y*‐axis reflects the average local density in occupied plots. Because density was averaged across multiple years, the maximum *x*‐value is the maximum occupancy achieved over the years under investigation (Supporting information Appendix S2).

We used randomization tests to determine whether total abundance shifted over time at each site. Specifically, we randomly assigned local densities to a time period, calculated the area between the two ROAPs (D*; Collins et al., 2009) 999 times, then compared the empirical D* to this distribution. We considered the change in abundance statistically significant if the empirical value for D* was among the 50 most extreme values.

## 3 RESULTS

### 3.1 Overview

**Figure 3:**
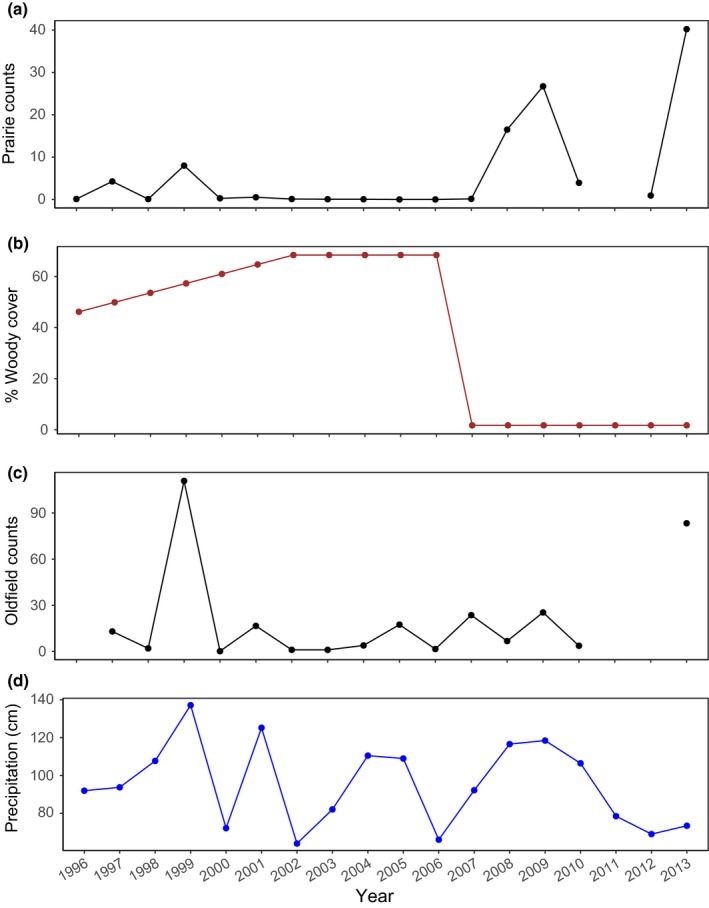
Yearly changes in numbers of *Agalinis auriculata *at prairie and old field sites, percent woody cover, and precipitation. (a) Average number of plants/plot for the prairie (excluding 2011); (b) Average percent woody cover/plot for the prairie (woody plants removed in 2006, after the survey). Negligible woody cover in the oldfield is not shown; (c) Average number of plants/plot for the oldfield (excluding 1996, 2011, and 2012; some years estimated, Supporting information Appendix S1); (d) Precipitation (cm)

Numbers of *A. auriculata* plants varied widely at both sites, both temporally (Figure 3) and spatially (Supporting information Figure S1). Plots that had most plants in 1 year were not always the plots that had most plants in other years (Supporting information Figure S1). Some years had relatively few plants at both sites (2010) while other years had high numbers (2009, 2013), suggesting some similarity in factors affecting dynamics across sites (Figure 3; Supporting information Figure S1).

In contrast to the oldfield, the prairie had extremely few plants between 2001 and 2007 (Figure 3, Supporting information Figure S1). Except for 2007, these years also had high woody cover (Figures 2a, 3). The average amount of woody cover at the prairie in 2002–2006, for example, was 68.4% (*SE *= 3.9%), and 44% of the plots had >80% woody cover. The plots with lowest woody cover in these years were often plots with a high percentage of eroded soil (Figure 2). The oldfield had very little woody cover (Figure 2); only 2 plots had >10% in any year.

**Figure 4:**
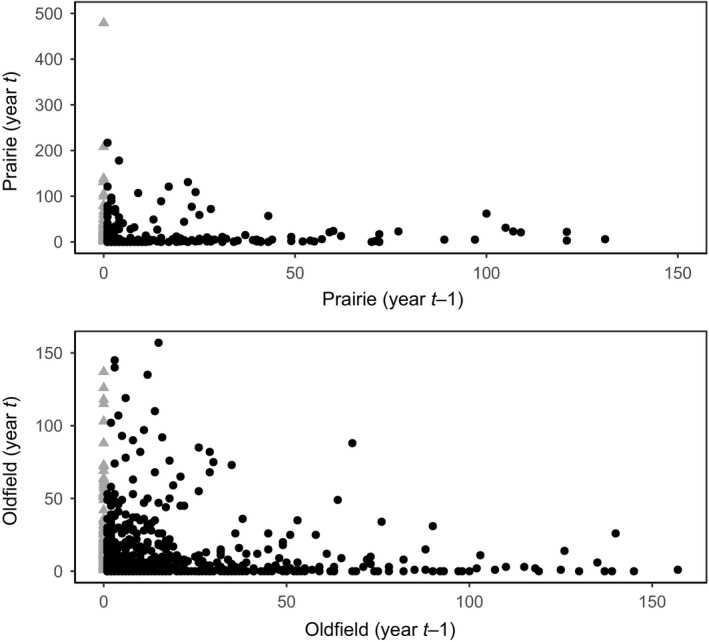
Plots of numbers of *Agalinis auriculata *in time *t* versus numbers in time *t *− 1 for 100 m^2^ plots in the prairie (a) and oldfield (b) excluding plots without plants in both years. Recruits are triangles (0 plants in year *t *− 1, >0 plants in year *t*). For the prairie, no data are available for 1995 or 2012, since no data were collected in 1994 or 2011. For the oldfield, the plot includes data only from 2001 to 2002 through 2009–2010 (pairs of years where all 119 plots were sampled).

For both prairie and oldfield, plots that had few plants in 1 year typically had many plants in subsequent years and vice versa (Figure 4a,b).

### 3.2 Average number of plants/plot: across years

Average number of plants/plot was marginally higher in the oldfield than the prairie for all years where data were available (paired *t* test, *t*_14_ = 1.94, *p* = 0.072); there was no difference after woody cover was removed (although sample size was low; paired *t* test, *t*_4_ = 1.13, *p* = 0.320). For the prairie, the average number of plants/plot was best predicted by average percent woody cover. The model with the lowest AIC only included woody cover, with higher plant number associated with lower woody cover: (log *N_t_* = 0.90–0.03 average percent woody cover; Figure 3). In contrast, the best fitting model for the oldfield suggested that average numbers of plants/plot increased with precipitation and if the previous year had high numbers, the subsequent year had lower numbers (log *N_t_* = −0.31 + 0.14 precipitation – 0.39 log *N_t_*_−1_; Figure 3).

For the years with shared data, the average number of plants/plot at the prairie and oldfield were positively correlated (Spearman's rank correlation, *n* = 15, *r_s_* = 0.54, *p* = 0.037).

### 3.3 Number of plants/plot: within years (prairie)

Prior to woody removal, greater woody cover was associated with fewer plants (2 of 3 years) and a higher percentage of eroded soil was associated with fewer plants (1 of 3 years; Table 1A). The models consistently showed a positive association between numbers in the previous year and numbers in the current year. Usually, the number of neighbors (previous or current year) were negatively associated with plant numbers.

**Table 1:** Parameter estimates (and *SE*) from best‐fit models describing how numbers of *Agalinis auriculata* in 66 prairie plots (each 100 m^2^) depend on percent woody cover, percent eroded soil, numbers of plants in the previous year, neighbors (numbers of plants in the 8 plots surrounding the focal plot), and neighbors in the previous year

A. Before woody removal			
	1997	1999	2001
	*N* = 282	*N* = 528	*N* = 35
Intercept	2.4716 (0.11)	3.9838 (0.15)	1.4659 (0.47)
Woody	0.0067 (0.003)	−0.0244 (0.002)	−0.0089 (0.006)
Eroded	–	−0.0469 (0.005)	–
Numbers*_t_*_−1_	0.3208 (0.08)	1.8373 (0.1)	0.5253 (0.18)
Neighbors*_t_*	–	−0.0039 (0.001)	−0.2612 (0.07)
Neighbors*_t_*_−1_	−0.4397 (0.07)	−0.1457 (0.04)	0.1547 (0.09)

B. After woody removal					
	2008	2009	2010	2012	2013
	*N* = 1,089	*N* = 1,764	*N* = 260	*N* = 62	*N* = 2,654
Intercept	2.5774 (0.06)	2.5377 (0.05)	0.3557 (0.14)	0.8000 (0.2)	2.7063 (0.04)
Woody	–	−0.0255 (0.007)	–	−0.0750 (0.04)	−0.0192 (0.005)
Eroded	−0.0874 (0.01)	−0.1000 (0.01)	–	0.0200 (0.004)	−0.0189 (0.005)
Numbers*_t_*_−1_	0.1158 (0.04)	–	0.0172 (0.002)	–	−0.1094 (0.02)
Neighbors*_t_*	0.0026 (0.003)	0.0037 (0.003)	−0.0326 (0.007)	–	0.0024 (0.0005)
Neighbors*_t_*_−1_	−0.0170 (0.007)	–	0.0065 (0.001)	–	–

Notes

*N* = total number of plants of *A. auriculata* in the year noted; years where the maximum number of plants in any one plot was ≤five were not analyzed. (A) Years before the fall 2006 removal of woody cover. (B) Years after the fall 2006 removal of woody cover.

After woody removal, increased woody cover was always negatively associated with plant numbers, though not included in all models (Table 1B). In 3 of 4 years, plots with more eroded soil were less likely to have plants. Depending on the year, plants in the previous year and number of neighbors (previous or current year) were positively or negatively associated with number of plants.

See Supporting information Table S1 for AIC values for the best and second best models for models in Table 1A,B, as well as ΔAIC values.

### 3.4 Comparison: 2001–2006 versus 2007–2010

#### 3.4.1 Plants/plot

Overall, there was greater consistency in model structure between the prairie and oldfield for 2007–2010 compared to 2001–2006 (Table 2). Specifically, precipitation was always positively associated with numbers; precipitation in the previous year was negatively related to number of plants in both sites from 2007 to 2010 (Table 2). The number of plants/plot in the previous year was positively associated with numbers while number of plants in neighboring plots (previous or current year) were generally positively associated with numbers for 2007–2010. One parameter (neighbors*_t_*_−1_) was, however, included in the model for the prairie but not the oldfield.

**Table 2:** Parameter estimates (and *SE*) from best‐fit models describing how numbers of *Agalinis auriculata* in 100 m^2 ^plots depend on percent woody cover, numbers of plants in the previous year, neighbors (numbers of plants in the 8 plots surrounding the focal plot), and neighbors in the previous year

	Prairie	Prairie	Oldfield	Oldfield
	2001–2006	2007–2010	2001–2006	2007–2010
Intercept	−2.0901 (1.69)	−6.0165 (0.72)	−2.0183 (0.28)	0.2873 (0.6)
Precipitation*_t_*	0.0262 (0.01)	0.0816 (0.007)	0.0205 (0.002)	0.0218 (0.008)
Precipitation*_t_*_−1_	–	−0.0119 (0.002)	0.0201 (0.002)	−0.0113 (0.004)
Numbers*_t_*_−1_	0.1840 (0.10)	0.0035 (0.001)	0.0177 (0.001)	0.0070 (0.003)
Neighbors*_t_*	−0.1411 (0.06)	0.0036 (0.0005)	0.0040 (0.0001)	0.0063 (0.0004)
Neighbors*_t_*_−1_	–	0.0009 (0.0001)	−0.0030 (0.0004)	–

Note

Models used either 2001–2006 data (years prior to woody removal at the prairie site) or 2007–2010 data (years after woody removal at the prairie site).

There was less consistency in patterns when comparing the prairie and oldfield from 2001 to 2006. Two parameters (precipitation and neighbors in the previous year) were included in the oldfield model but not the prairie model. For numbers*_t_*_−1_, the term was negative for the prairie and positive for the oldfield, although both precipitation and numbers in the current year were both included in the final model and both had positive coefficients.

See Supporting information Table S2 for AIC values for the best and second best models for models in Table 2, as well as ΔAIC values.

#### 3.4.2 Abundance and occupancy

**Figure 5:**
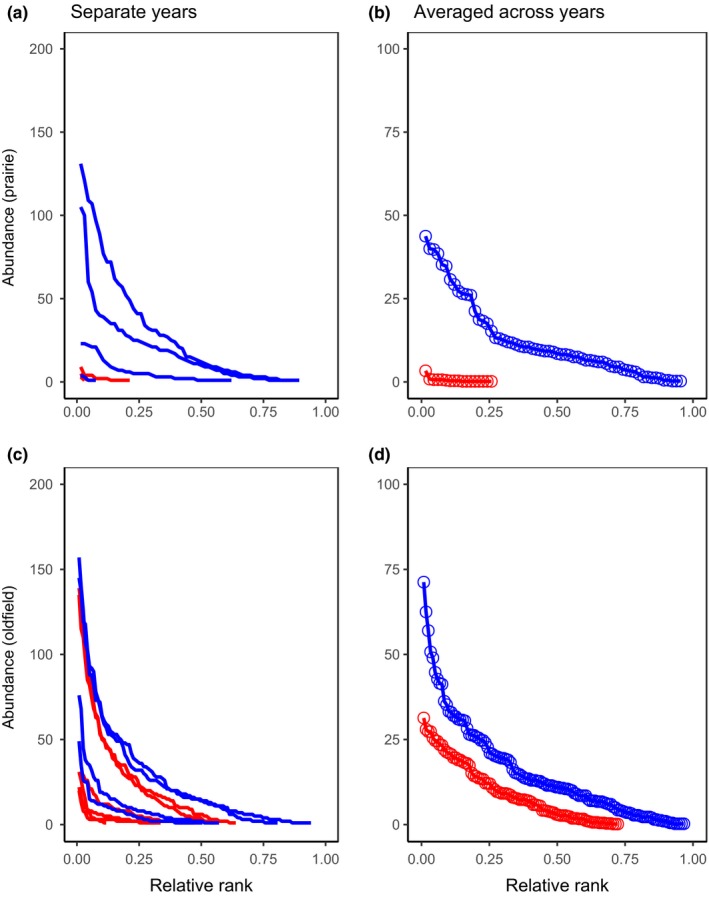
Rank occupancy‐abundance profiles (ROAPs) for *Agalinis auriculata.* ROAPs were constructed for (a) prairie (individual years), (b) prairie (averaged across years for two time periods: before woody removal in the prairie (red, 2001–2006) and after woody removal in the prairie (blue, 2007–2010), (c) oldfield (individual years), and (d) oldfield (averaged across years for two time periods: before woody removal in the prairie (red, 2001–2006) and after woody removal in the prairie (blue, 2007–2010). For plots of individual years, local abundance was measured as numbers of plants in a 100 m^2^ plot (*y*‐axis) and the *x*‐axis refers to the relative rank (i.e., a plot with the highest abundance has the lowest relative rank). The highest *Y* value for a ROAP indicates the maximum number of plants per plot, and the remaining *Y* values on the line reveal successively smaller plot abundances. The x‐intercept of a ROAP for separate years indicates proportion of plots with plants (e.g., occupancy). For (a) and (c), individual plot values are not shown to improve clarity. See Supporting information Appendix S2 for explanation of how ROAPs are created, including interpretation of occupancy for ROAPs averaged over years

There was considerable year‐to‐year variability in abundance and occupancy at the prairie both before (2001–2006) and after (2007–2010) woody removal (Figure 5a; Supporting information Figure S1). Overall abundance of plants across the prairie increased over 8,500% ([recent number–original number]/original number) following woody removal (D* = 771.75, *p* < 0.001; Figure 5b). This large % increase is because the prairie originally had very few plants. We observed concurrent increases in local density and spatial expansion: occupancy increased from 0.06 (±0.03 *SE*) to 0.61 (±0.19); Figure 5a,b.

The overall abundance of plants across the oldfield saw an increase of 114% over these same time periods (before: 2001–2006, after: 2007–2010; Figure 5c,d). This increase was statistically significant (D* = 942; *p* < 0.001). Average occupancy in the oldfield increased from 0.41 (±0.08) to 0.71 (±0.10).

Analyses performed with the prairie data for all years (before: 1996–2006, after: 2007–2013, excluding 2011) showed similar increases in abundance and occupancy (Supporting information Figure S2, D* = 889.53, *p* < 0.001).

## 4 DISCUSSION

### 4.1 Factors affect plant population numbers

Our long‐term study of the rare annual *Agalinis auriculata *revealed that woody colonization and removal were key factors affecting numbers. Average woody cover was the only predictor of average number of plants per prairie plot over a 17‐year period, with plant numbers declining as woody cover increased*.* Prairie plots with higher woody cover had fewer *A. auriculata *plants in most years. We also note that both prairie and oldfield had more similar population models after woody removal than before, suggesting that dynamics at the two sites converged after woody removal. Finally, although plot occupancy and plant abundance were higher after than before woody removal at both sites, the magnitude of increase over time was much greater for the prairie (in part because it was so rare prior to tree removal). Overall, these results suggest that the *A. auriculata *prairie population was constrained by *J. virginiana *in the first half of our study.

Vitt et al. (2009) proposed that increases in *A. auriculata *after woody removal would be temporary because competitive graminoids also increase in open sites. At our prairie, our last survey year (7 years after woody removal) had unusually large numbers of *A. auriculata *plants, suggesting a longer‐term effect of woody removal. However, the general point that vegetation will change over time following management is important. Limb et al. (2014), for example, noted successional trends in a plant community over 5 years after *J. virginiana* removal, with increasing dominance of herbaceous perennials.

We lack data on what factors affected woody colonization at the site, as well as the mechanism for decline in *A. auriculata *when *J. virginiana* became common*. Agalinis auriculata *was likely shaded out by trees, but water availability or soil properties could also have been altered. Given that *A. auriculata *is a hemiparasite, another possibility is that the high tree cover reduced host numbers, although probable hosts (Asteraceae; Molano‐Flores et al., 2003) were present at both sites and over time.

Other exogenous factors also were associated with plant numbers, especially precipitation. Not surprisingly, high precipitation years had high plant numbers. We do not know in detail how precipitation affected plant survival or reproduction; in some annuals, for example, the timing of rain was more important than total annual rainfall for population dynamics (Levine et al., 2011). Different factors may also interact: Figure 3 shows that the years immediately following woody removal also had high rainfall, likely facilitating population growth at the prairie. Another exogenous factor at the prairie was the percentage of eroded soil; high erosion plots typically had fewer plants of *A. auriculata *(few plants of any species grow in these sites).

Endogenous processes, including negative density‐dependence, are often important in plant population dynamics (Crawley, 1990; Garcia de Leon et al., 2014; Gonzalez‐Andujar et al., 2006; Plaza et al., 2012). Consistent with this concept, plots with large numbers of plants in 1 year had smaller numbers of plants in the next year and vice versa*. *Using the average plant data set, the oldfield model has a positive term for precipitation and a negative coefficient for previous year numbers. Perhaps high water availability contributed to an increased population size, but plants at high density had reduced per‐capita seed production. Multiple mechanisms could be at work: intraspecific competition, disease spread at high densities, or concentrated herbivore feeding in high‐density plots are possibilities.

We did not directly test for density‐dependence because our local data set included plots without plants (zero density; no possibility for density‐dependent processes). Interestingly, the coefficient for previous year numbers was often positive in these models, perhaps suggesting that the presence of plants in past years was indicative of conducive locations for plant growth in future years. These positive coefficients may also be a byproduct of using zero‐inflated binomial distributions, which in effect may reduce the role of the zeroes in the analyses.

Plots with zero plants in 1 year sometimes had many plants present in subsequent years. These recruits could be from seed dispersal, from germination from dormant seeds (Baskin et al., 1991) or could reflect observer error (lack of detection of plants in the first year or slight yearly variation in delineation of plot boundaries). Seed bank germination seems most likely for the 2012–2013 recruits (2012–2013 transitions were over 1/4th of the prairie plots with 0 plants in 1 year and >0 in the next year, including the five extreme cases with more than 130 plants in 2013). 2012 was unusually dry and had very high summer temperatures; we speculate that few seeds germinated in 2012, but did germinate in 2013. Similar patterns of 100’s – 1,000’s of seedlings appearing in sites with zero plants the year before occurred in studies of annual sunflower, another seed bank species (Alexander et al., 2009).

Although we expect that seed banks, as opposed to seed dispersal, are the most likely explanation for transitions from zero plants/plot to 100’s of plants/plot, as noted above, seed dispersal is also likely ecologically important. We were surprised to find *A. auriculata *at the oldfield site in 1997 given that the area had been tilled in 1995 (Supporting information Appendix S2); presumably these plants are the result of dispersal from the nearby prairie. We do not know the degree to which the numbers of plants at the oldfield in later years were derived from dispersal from the prairie or a result of seed production at the oldfield site.

On a small spatial scale, we also explored the role of seed dispersal when we added the number of neighbors to models to include some spatial structure because we expected that neighbors in a previous year could disperse seed into a plot and thus be positively associated with plant numbers the next year. Further, plots near each other might have similar environments and thus we predicted that plant numbers in neighboring plots would be positively associated with each other within the same year. In fact, we saw both negative and positive coefficients for both neighbors*_t_* and neighbors*_t_*_−1 _in models. There was a tendency to see more negative values before woody removal in the prairie. The patchy nature of woody cover could have meant that plots with *A. auriculata *plants were often surrounded by plots with high tree cover.

Herbivory was not measured but could contribute to variation in plant numbers and, like precipitation, to similarity in fluctuations for adjacent sites. Deer, small mammals, and insects all feed on *A. agalinis* (Mulvaney, Molano‐Flores, & Whitman, 2006; Packard, 2012; Vitt et al., 2009). Estimated deer densities at the KU Field Station ranged from 0.04 to 0.18/ha from 2008 to 2013 (R. Hagen, unpublished data); a year with very high plant numbers (2013) had the lowest deer density. Ward (1994) noted that caterpillars of *Junonia coenia* (buckeye butterfly) killed nearly all *A. auriculata* at our site in a year prior to our study. Herbivore numbers may increase regionally due to other food plants with negative effects on rare hosts like *A. auriculata *(apparent competition; Holt & Bonsall, 2017)*.*

### 4.2 Rarity, restoration, and roaps

In addition to studying *A. auriculata *in a remnant prairie, our work in a post‐agricultural field provides data relevant to restoration. Specifically, although known as a rare plant, *A. auriculata *colonized an oldfield and persisted with often large population sizes. This species thus has a tolerance of disturbed sites, and potentially could be introduced into prairie restoration plantings, especially since Asteraceae (hosts for this hemiparasite) are common in prairie seed mixes. As noted previously, we found that seeds can at least sometimes disperse large distances (i.e., meters as opposed to centimeters), suggesting a “spillover” effect where a desirable species disperses from a remnant site (prairie) into non‐target habitat (oldfield; Brudvig, Damschen, Tewksbury, & Haddad, 2009).

Like most ecological studies, our data are site and species‐specific.  However, our work suggests three general lessons for management of herbaceous grassland species where woody colonization is occurring.  First, we show that improvement in population trends for a rare species is possible with tree cutting and without reseeding: too often managers may not take any action because they feel any efforts are hopeless. Second, as perhaps obvious, long‐term records are essential, given that current threats (woody colonization) and management responses (woody removal) occur over many years. Our results (Figure 3) reveal the tremendous fluctuation in numbers that can occur across years for annual species, illustrating that results from any 2–3 year period could lead to misinterpretations.  Third, we note that by taking data with a relatively simple method (number of plants in large plots), we could extensively sample two adjacent habitats (prairie and oldfield). If we had only taken data in a subset of the area using small plots, we could easily have missed major trends, especially because plants “moved around” both habitats over the years (Supporting information Figure S1).

Haddad et al. (2008) suggest that optimal monitoring schemes for rare butterflies require consideration of the need for information, statistical performance, and the costs of different approaches. These same factors must be considered with plants. With annuals, count data, such as presented here, have logistical advantages. Annual surveys required only 2–3 days with an established gridwork. Of course, combining demographic and survey approaches would be ideal and allow greater understanding of the mechanisms leading to changes in numbers of plants. For example, plant size and reproduction data would have allowed us to compare our work to Vitt et al. (2009) who noted that the proportion of large individuals (who produce the most seed) increased with woody removal. Regardless of monitoring approach, it is obvious that one needs consistency in data collection methods across years and careful record‐keeping. In our case, work had been largely done by the same people for the 17 year period, but that is rarely the case and many long‐term studies are doomed by poor data quality.

ROAPS are another tool to consider in optimal monitoring schemes. Although not spatially explicit, ROAPS provides insights into spatio‐temporal shifts in abundance that may not otherwise be apparent. It is also important to emphasize that many land managers have limited time, and monitoring schemes does not always require the most advanced tools. Simple monitoring of local plant densities and spatial spread, as done in this study, can be used to plot ROAPs (single year or averaged over years, Supporting information Appendix S2) to compare abundances over time, between landscape types, or before/after management actions. Such visual tools allow land managers to discern whether overall abundance is shifting due to changes in local density, spatial extent, or both, and thus more readily use past data to guide future management decisions. For instance, ROAPs could be useful for invasive plant research: management responses would likely differ if occupancy increased more rapidly over time than did local abundance, as opposed to situations where the plant was highly abundant locally but not spreading.

## CONFLICT OF INTEREST

None declared.

## AUTHOR CONTRIBUTIONS

WDK, HMA, and CDC conceived ideas and designed methodology; VBS, WDK, HMA, and DAC collected data; AWR, CDC, HMA, WDK, DAC, and LDC analyzed data with AWR leading on general linear model analyses and CDC leading on ROAPs analyses; HMA and CDC led writing of the manuscript. All authors contributed critically to drafts and gave final approval for publication.

## DATA ACCESSIBILITY

Data available from the Dryad Digital Repository: https://doi.org/10.5061/dryad.801r9q8.
