# Supplementary material for: Effects of removing woody cover on long‐term population dynamics of a rare annual plant (Agalinis auriculata): A study comparing remnant prairie and oldfield habitats
Source: Ecol Evol. 2018 Nov 11;8(23):11975–86. doi: 10.1002/ece3.4654 (PMC6303752; doi:10.1002/ece3.4654)

**Appendix S1. Additional details about the prairie and oldfield study sites and analyses**

*Prairie history and management*

Historical aerial photographs of the prairie dating back to 1937 reveal several patches of severely eroded soil with high clay content (Dickey et al., 1977) that have persisted for decades.  These patches are sparsely vegetated likely due to the strongly expressed shrink-swell properties of the exhumed subsurface soil horizons which can impede root growth (Bengough et al., 2006).  A similar mechanism was used to explain the concentration of roots in the upper 4 cm of an eroded soil in a similar landscape position approximately 1.2 km from this site (Hirmas et al., 2013).

From 1971 until tree removal in 2006, the prairie had no management or agricultural use. Given past fencing, it was likely used as pasture at times before 1971. Ward (1994) studied the site from 1991-1993. Ward’s work was primarily observational, although he did do small scale experiments (1991; thirty six 50 cm x 50 cm plots, with one third assigned to a control treatment, one third to a close mowing treatment, and one third to a soil tilling treatment; 1992, 18 of the 50 cm x 50 cm plots from 1991 had seed from the same site added as part of seed limitation study).

Since 1971, colonization of eastern red cedar (*Juniperus virginiana* L. (Cupressaceae) is increasingly evident in aerial photographs. In the mid 2000s, managers decided to remove woody vegetation given concerns of adverse effects on herbaceous prairie plants, including *A. auriculata.*  After plant surveys in fall 2006, all woody vegetation (except small shrubs) was cut at ground level and stacked in brush piles outside the prairie along the 3 m vehicle trail separating the prairie and oldfield. Over 90% of the cut plants were *J. virginiana.* The prairie was burned in the spring of both 2009 and 2010.

*Oldfield history and management*

The oldfield site had been plowed and was likely planted to crops for decades before 1970. In 1985, 1986, and 1988 the field was tilled and planted to cover crops and it was mowed in 1989 and 1994. In 1995, the field was tilled and used for a field experiment with *Helianthus annuus*. The site then had no management from 1996-2006, except in late 2006 (cut trees from prairie piled along the path and on parts of the field). In January 2007, the oldfield was mowed. The field (including piles of cut woody vegetation) was burned in spring 2009 and 2010 along with the prairie site and the site was mowed in summer 2012. Due to site history and management practices, only very limited woody plant colonization occurred in the oldfield.

*Vegetation history at the prairie and oldfield sites*

We lack comprehensive vegetation data over the 1996-2013 time period. Lists of plant species found at the site in 1989-1990 reveal that 98 species were found in the prairie and 48 species at the oldfield site. The prairie grass, *Sporobolus asper*, and sedges were common at the prairie and many prairie forbs were observed (e.g., *Solidago rigida, Lespedeza capitata, Silphium lacinantum*). In contrast, the oldfield site was dominated by weedy species such as *Ambrosia artemisiifolia* and *Setaria viridis*, and similar vegetation would be expected after tilling in 1995. Ward (1994) estimated percent cover in thirty 0.1 m^2^ quadrats at the prairie site and found 42 species. The most common species were grasses (*Sporobolus asper*, *Dichanthelium* spp.), species in the Asteraceae (*Ambrosia artemisiifolia, Antennaria neglecta*, *Aster pilosus*, and *Helianthus rigidus*)*,* as well as *Acalypha* spp. and *Polygala verticillata*. Ashworth et al. (2010) established six 5 m x 5 m plots in the prairie site in 2005 and estimated percent cover of species in July 2005, June 2008, and June 2010. Years after the 2006 removal of woody plants were associated with increased percent cover of native forbs and grasses (29.9%, 2005; 156.7%, 2008; 140.5% 2010; numbers exceed 100% because of 3D layering of foliage. Species richness also increased: 44 (2005), 81 (2008), and 73 (2010). In 2005, Ashworth et al. (2010) noted the prairie site was dominated by *Sorghastrum nutans, Schizachyrium scoparium,* and *Liatris squarrosa*. *Dichanthelium acuminatum* and *Ambrosia artemisiifolia* were particularly common in, respectively, 2008 and 2010. We lack data on the oldfield site after 1989-1990 but after tilling ceased in 1995, succession occurred at the site and prairie grasses colonized. Four years after the study (Fall 2017), the dominant vegetation at both prairie and oldfield sites were prairie grasses (*Sorghastrum nutans*, *Schizachyrium scoparium*).

*Calculations for the average plant data set*

To explore dynamics over the longest period of time, we analyzed the average number of plants per plot per year (average plant data set). For the prairie, we calculated this variable by summing all plants seen in a year and dividing it by the number of plots (66) (such values are available 1996-2013 (excluding 2011). For the oldfield, we took this same approach for 2001- 2010 (summing all plants seen in a year and dividing by 119, the number of plots). From 1997-2000, only six oldfield plots were sampled, so we added up the total number of plants in these plots and divided by 6. This approach seemed reasonable because the average number of plants per plots for the six plot subset was positively correlated with the remaining 113 oldfield plots for the 2001-2010 period. Similarly, for 2013, we summed up the number of plants in the 14 sampled oldfield plots and divided by 14. Note that this data set did not include plots that were intercepted by the vehicle path or that had woody brush piles on them for some years. The average plant data set had 16 (prairie) or 15 (oldfield) rows of data.

References:

Ashworth, S., Kettle, W. D., Dobbs, K. E. 2010. Response of herbaceous plant community after removal of woody vegetation in a tallgrass prairie restoration. 22^nd^ North American Prairie Conference. Pp. 24-30.

Bengough, A.G., Bransby, M.F., Hans, J., McKenna, S.J., et al. 2006. Root responses to soil physical conditions; growth dynamics from field to cell. J. Exp. Bot. 57, 437–447.

Dickey, H.P., Zimmerman, J.L., Rowland, H.T., 1977. Soil Survey of Jefferson County, Kansas. U.S. Dep. of Agric., Soil Conservation Service in cooperation with the Kansas Agric. Exp. Station. U.S. Gov. Print. Office, Washington, DC.

Hirmas, D.R., Giménez, D., Subroy, V., Platt, B.F., 2013. Fractal distribution of mass from the millimeter- to decimeter-scale in two soils under native and restored tallgrass prairie. Geoderma 207-208, 121-130.

Ward G. 1994. Ecology of *Tomanthera auriculata*, a rare annual plant. Master’s Thesis, Univesity of Kansas, Lawrence, KS

**Table S1** AIC values for the best and second best models in Tables 1A and 1B, as well as ΔAIC values.

|  | AIC | ΔAIC |
| --- | --- | --- |
| **1997** |  |  |
| Woody Numbers­­_t-1_ Neighbors­_t-1_ | 413.6 |  |
| Numbers­­_t-1_ Neighbors­_t-1_ | 437.9 | 24.3 |
| **1999** |  |  |
| Woody Eroded Numbers­­_t-1_ Neighbors­ Neighbors­_t-1_ | 375.6 |  |
| Woody Eroded Numbers­­_t-1_ Neighbors­_t-1_ | 380.2 | 4.6 |
| **2001** |  |  |
| Woody Numbers­­_t-1_ Neighbors Neighbors_t-1_ | 110.4 |  |
| Woody Eroded Numbers­­_t-1_ Neighbors Neighbors_t-1_ | 112.5 | 2.1 |
| **2008** |  |  |
| Eroded Numbers­­_t-1_ Neighbors Neighbors_t-1_ | 1305 |  |
| Numbers­­_t-1_ Neighbors Neighbors_t-1_ | 1349 | 44 |
| **2009** |  |  |
| Woody Eroded Neighbors | 1189 |  |
| Woody Eroded Neighbors Numbers­­_t-1_ | 1197 | 12 |
| **2010** |  |  |
| Neighbors Numbers­­_t-1_ Neighbors_t-1_ | 313.4 |  |
| Neighbors Numbers­­_t-1_ Neighbors_t-1_ Eroded | 326.6 | 13.2 |
| **2012** |  |  |
| Woody Eroded | 140.0 |  |
| Woody Eroded Numbers­­_t-1_ | 147.2 | 7.2 |
| **2013** |  |  |
| Woody Eroded Numbers­­_t-1_ Neighbors­ | 2096 |  |
| Woody Eroded Numbers­­_t-1_ Neighbors­ Neighbors_t-1_ | 2099 | 3 |

**Table S2** AIC values for the best and second best models in Table 2, as well as ΔAIC values.

|  | AIC | ΔAIC |
| --- | --- | --- |
| **Prairie 2001-2006** |  |  |
| Precipitation Numbers­­_t-1_ Neighbors_t-1_ | 215.16 |  |
| Numbers­­_t-1_ Neighbors_t-1_ | 228.1 | 12.94 |
| **Prairie 2007-2010** |  |  |
| Precipitation Precipitation_t-1_ Numbers­­_t-1_ Neighbors Neighbors_t-1_ | 3126 |  |
| Precipitation Numbers­­_t-1_ Neighbors | 3137 | 11 |
| **Oldfield 2001-2006** |  |  |
| Precipitation Precipitation_t-1_ Numbers­­_t-1_ Neighbors Neighbors_t-1_ | 3404 |  |
| Precipitation, Numbers­­_t-1_ Neighbors Neighbors_t-1_ | 3575 | 171 |
| **Oldfield 2007-2010** |  |  |
| Precipitation Precipitation_t-1_ Numbers­­_t-1_ Neighbors | 2783 |  |
| Precipitation Numbers­­_t-1_ Neighbors | 2788 | 5 |

**Figure S1**

Spatial patterns of *Agalinis auriculata* at the prairie and oldfield sites in northeastern Kansas, USA. Orientation matches Figure 1, with the prairie to the upper left and the oldfield to the lower right. North is to the top of the graph. Graphs are bubble plots based on censuses in 100 m^2^ plots from 1996 – 2013. A circle is shown if plants are present in a plot; circles are consistently scaled across all sites and years; the largest circle reflects the largest number ever seen at the site (479 observed in 2013 in the prairie). No data are shown for plants occupying the vehicle path between the prairie and oldfield. As described in the text, complete censuses were not done in the oldfield over all years; years without censuses or with incomplete censuses are indicated on the appropriate figures.

Fig. S2. Rank occupancy-abundance profiles (ROAP’s) for *Agalinis auriculata* for the prairie using all data available. ROAPs were constructed for A) individual years and B) averaged across years for two time periods: before woody removal (red, 1996-2006) and after woody removal (blue, 2007-2013, excluding 2011 when surveys were not conducted). For plots of individual years, local abundance was measured as number of plants in a 100 m^2^  plot (y-axis) and the x-axis refers to the relative rank (i.e., a plot with the highest abundance has the lowest relative rank). The highest Y value for a ROAP indicates the maximum number of plants per plot, and the remaining Y values on the line reveal the successively smaller plot abundances. The x- intercept of a ROAP for separate years indicates the proportion of plots with plants (e.g., occupancy). Note that for A), individual plot values are not shown to improve clarity. See Appendix S2 for explanation of how ROAPs are created, including interpretation of occupancy for ROAPs averaged over years.


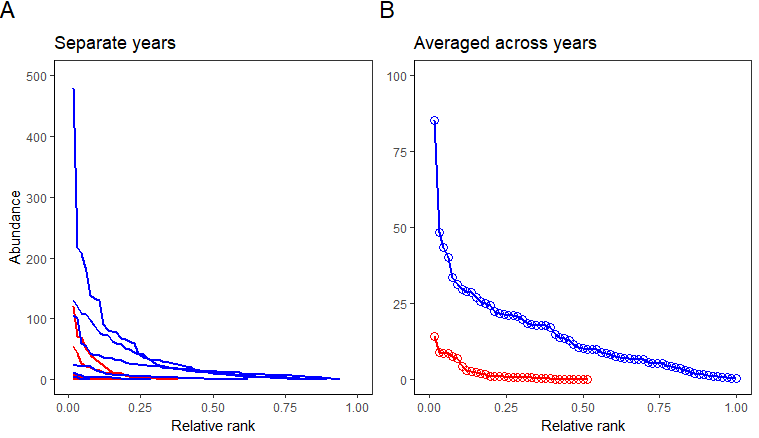

Supplement: Supplementary file 1 [file ECE3-8-11975-s001.docx]
